# Blockade of the Formation of Insoluble Ubiquitinated Protein Aggregates by EGCG3”Me in the Alloxan-Induced Diabetic Kidney

**DOI:** 10.1371/journal.pone.0075687

**Published:** 2013-09-30

**Authors:** Shuxian Cai, Yuan Zhong, Yinhua Li, Jianan Huang, Jing Zhang, Guoan Luo, Zhonghua Liu

**Affiliations:** 1 Key Laboratory of Ministry of Education for Tea Science, Hunan Agricultural University, Changsha, Hunan Province, China; 2 Department of Biochemistry and Molecular Biology, Peking University Health Science Center, Beijing, China; 3 National Research Center of Engineering Technology for Utilization of Botanical Functional Ingredients, Changsha, Hunan Province, China; 4 Department of Chemistry of Tsinghua and Key Laboratory of Biological Organic Phosphorus and Chemical Biology of Ministry of Education, Tsinghua University, Beijing, China; University of South Florida College of Medicine, United States of America

## Abstract

**Background:**

Renal accumulation of reactive carbonyl compounds (RCCs) has been linked to the progression of diabetic nephropathy. We previously demonstrated that carbonyl stress induces the formation of amino-carbonyl cross-links and sharply increases the content of β-sheet-rich structures, which is the seed of insoluble aggregates formation, and tea catechin (-)-epigallocatechin 3-gallate (EGCG) can reverse this process in vitro and in vivo. In this study, methylated derivative (-)-epigallocatechin-3-*O*-(3-*O*-methyl)-gallate (EGCG3”Me) was hypothesized to neutralize carbonyl stress mediating the formation of insoluble ubiquitinated protein (IUP) aggregates, and reduce the early development of diabetic nephropathy.

**Methods and results:**

Diabetes was induced in mice by intraperitoneally injecting alloxan monohydrate (200 mg/kg/d) twice and administering EGCG3”Me by gavage for 15 d. Reagent case and western blot results showed that, in diabetic kidneys, the carbonyl proteins in the serum increased; and in insoluble protein fraction, 4-hydroxynonenal-modified proteins, IUP aggregates and p62 accumulated; FT-IR study demonstrated that the lipid content, anti-parallel β-sheet structure and aggregates increased. EGCG3”Me treatment could effectively reverse this process, even better than the negative control treatment.

**Conclusions:**

EGCG3”Me exhibiting anti-β-sheet-rich IUP aggregate properties, maybe represents a new strategy to impede the progression of diabetic nephropathy and other diabetic complications.

## Introduction

The accumulation of insoluble ubiquitinated proteins (IUP) that are deposited intracellularly is related to the pathogenesis of age-related chronic diseases, such as obesity, diabetes, and cardiovascular and renal disorders [1]. p62/Ref(2)P family of proteins is involved in the delivery of ubiquitinated protein aggregates to autophagosomes. However, impaired autophagy of defects in the protein degradation pathway results in the accumulation of the formation of p62 and ubiquitinated protein aggregates because of the nature of both self-oligomerization and ubiquitin binding of p62 [2]. Protein aggregation leads to the accumulation of intracellular ubiquitin conjugates and cell cycle arrest, which is a potential mechanism associating protein aggregation to cellular dysregulation and cell death [3], [4], [5].

β-sheet structure is the seed of aggregate formation characterized by the slow folding of an intermediate state of a transient helix and the rapid folding from the nucleation of a β-turn [6]. β-sheet is important in biological functions and malfunctions because an aggregate of β-sheets forms a β-barrel that transports ions and small molecules (and removes unwanted molecules) across the cell membrane [7]. Hydrophobic interactions are important to hold different β-sheets together and are thus essential features of the aggregation process. Therefore, molecules that can bind to the hydrophobic region but do not form β-sheets by themselves are currently developed and screened as drugs against diseases characterized by the formation of protein aggregates [8].

Our previous study showed that the amino-carbonyl cross-links induced by reactive carbonyl compounds (RCCs) can promote the formation of β-sheet structure [9]. RCCs formed during lipid peroxidation and sugar glycoxidation, namely advanced lipid peroxidation end products (ALEs) and advanced glycation end products (AGEs), form cross-links on tissular proteins, and accumulate during ageing and in chronic diseases. As a result, steady and irreversible cross-links of carbonyl-amino compounds have been found to be a common and essential toxiferous process, which might therefore be at the root of diseases and aging-related functional losses [10], [11]. (-)-epigallocatechine 3-gallate (EGCG) can reverse this process in vitro and in vivo. A structure-activity relationship study has suggested that galloyl D-ring is an important component that neutralizes the amino-carbonyl cross-linking reaction [9], [12]. In general, EGCG is considered as a potent remodeling agent of mature amyloid fibrils; EGCG also directly binds to β-sheet-rich aggregates and mediates the conformational changes [13]–[15].

**Figure 1:**
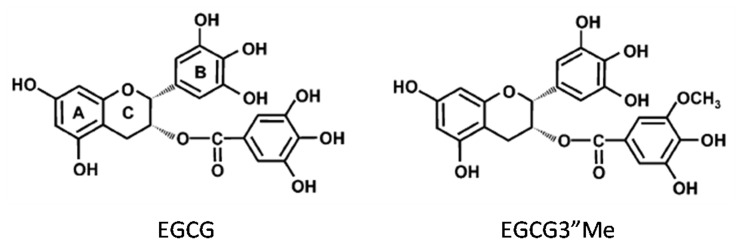
Chemical structures of EGCG and EGCG3”Me isolated from tea leaves. EGCG has flavan-3-ol structure with A and B rings and a D-galloyl group. EGCG3”Me contains a methyl ether group at the 3″ position of the D ring.

These findings have led to other questions, for instance, whether or not galloyl type of tea catechin is a possible inhibitory agent of β-rich aggregates in vivo. The structural and functional relationship of EGCG has shown that the hydroxyl groups on the 4′-position in the B ring and the 4″-position in the gallate are crucial for the cellular surface binding activity of EGCG. However, EGCG is known as unstable and can be degraded easily in animal bodies; the methylated derivative (-)-epigallocatechin-3-*O*-(3-*O*-methyl) gallate (EGCG3”Me) (Figure 1) is absorbed efficiently and more stable than EGCG in animal and human plasma; this substance also maintains the trihydroxyl structure of the B ring and the gallate structure that binds to the cell surface [16], [17]. *O*-methylated catechins are distinguished for their allergic affects [18].

In the present study, EGCG3”Me was used to study the protective effects and mechanism in diabetic mice. We successfully constructed an alloxan-induced diabetic mice model; the mechanism involves toxic glucose analogs that preferentially accumulate in pancreatic beta cells via GLUT2 glucose transporter [19]. This process increases the formation of reactive carbonyl compounds, such as AGEs. This study aimed to determine whether or not renal injury in diabetes is partly mediated by carbonyl stress-induced β-sheet misfold protein aggregates and EGCG3”Me as a delayed intervention. We also aimed to determine whether or not this result can improve renal structure and function once early renal injury becomes evident.

## Materials and Methods

### Ethical Statement

All animal studies were carried out in strict accordance with the recommendations in the Guide for the Care and Use of Laboratory Animals of the National Institutes of Health. The protocol was approved by the Ethics of Animal Experiments of the Peking University (Permit Number: SYXK (jing) 2011–0039). The animals were kept in a specific pathogen-free facility at Peking University Health Science Center, and all efforts were made to minimize the suffering of animals.

### Materials

EGCG3”Me (≥96%) and alloxan monohydrate were obtained from the Key Laboratory of Ministry of Education for Tea Science, Hunan Agricultural University and Sigma, respectively.

### Experimental Animal Model

Male C57BL/6 mice, approximately 7 weeks old and weighing about 20 g to 25 g, were obtained from the Charles River Laboratories (Beijing, China). The mice were kept in polycarbonated cages in a well-aerated room at 22±1°C with a 12 h/12 h light/dark cycle and fed with commercial pellets and water ad libitum [20]. The mice were allowed to adapt to the diet and general conditions in a vivarium for one week before the experiment was conducted. All of the experiments were performed in accordance with the institutional ethical guidelines.

Diabetes was induced by intraperitoneally injecting alloxan monohydrate [200 mg/kg body weight dissolved in sodium citrate buffer (pH 4.5)] twice at 48 h intervals. Sodium citrate buffer (pH 4.5) was injected in mice in the control group. One week after the second injection was administered, blood glucose levels (both random and fasting 6 h) were monitored by using blood glucose meter (Roche). The mice with blood glucose levels >16.4 mmol/L were considered diabetic and used for this study. Three doses (10, 20, and 30 mg/kg/d) of EGCG3”Me were administered. Diabetic and control mice were randomized into groups (*n* = 8). In the experiment, body weight and food intake of each group were recorded regularly. After 4 weeks of treatment, mice were killed under sodium pentobarbital anesthesia after feed-deprived for 12 h, and blood samples and kidneys were collected.

### Determination of Blood Glucose and Protein Carbonyl Contents

The whole blood glucose of each mouse was detected using an automatic biochemical analyzer (TOSHIBA). The whole bloods were centrifuged to collect the serum (at 3500 rpm and 4°C for 10 min), and Protein carbonyl content was detected using a carbonyl protein assay kit (Nanjing Jiancheng Bioengineering Institute, China).

### Westen Blot Analysis

Kidney samples were sonicated and resuspended in RIPA lysis buffer (Applygen Technologies Inc., Beijing, China) containing a protease inhibitor mixture (Fermentas). The mixture was lysed in an ice bath for 30 min and centrifuged for 15 min at 15,000×*g* at 4°C. The supernatant was collected, and the protein concentration was determined using a BCA protein assay reagent kit (Pierce). Total protein (50 µg to 80 µg) was subjected to 10% sodium dodecyl sulfate-polyacrylamide gel electrophoresis and transferred to nitrocellulose membranes (Millipore). These membranes were blocked in 5% non-fat dry milk in Tris-buffered saline with Tween (TBST; 10 mM Tris-Cl, pH 7.5, 150 mM NaCl, 0.05% Tween 20), washed four times with TBST, and incubated with horseradish peroxidase-conjugated secondary antibodies (Zhongshan Biotechologies Inc., China) for 1 h at room temperature [21]. The proteins were visualized using a chemiluminescent substrate (Millipore) according to the manufacturer’s instructions. The following antibodies were used for Western blot analysis: anti-RAGE (Santa Cruz Biotechnology); anti-IL6 (Santa Cruz Biotechnology); anti-p27 (Santa Cruz Biotechnology); and anti-GAPDH (Santa Cruz Biotechnology).

### Immunoblot Analysis of 4-HNE Modified and Ubiquitinated Protein Aggregates

The kidneys were placed individually in a 10 mL glass homogenizer with RIPA lysis buffer (Applygen Technologies Inc., Beijing, China) containing a protease inhibitor mixture (Fermentas). The mixture was lysed in an ice bath for 30 min and vortexed several times once every 10 min. Kidney lysates were centrifuged for 15 min at 12,000×*g* at 4°C. The supernatant was collected as the Triton-100 soluble fraction. The remaining pellet was resuspended in 10 mM Tris-HCl, 1% SDS for 10 min at room temperature. After centrifugation, the supernatant was collected as an insoluble fraction of Triton-100 [1], [22]. The protein concentration was determined using the BCA protein assay reagent kit (Piece). Western blot analysis was performed using the procedures described in Section (Westen blot analysis). The following antibodies were used: anti-4-HNE (Millipore); anti-multiubiquitin (MBL); anti-p62 (Epitomics); and anti-GAPDH (Santa Cruz Biotechnology).

### FTIR Analysis of Protein

Spectra were obtained at a reflectance mode ranging from 4000 cm^–1^ to 400 cm^–1^ at a spectral resolution of 4 cm^–1^, accumulating 32 scans per spectrum. Each sample was vacuum dried at −42°C for 24 h to remove the absorbed water in the kidney sample.

### IR Spectral Band Assignment Analysis

The maps were analyzed in Thermo Nicolet software OMNIC 6.0 by using raw, unprocessed spectra. Smoothing and derivatization were not performed to prevent contamination from subtle artifacts. The general assignment of IR bands in spectroscopy is well established. The asymmetric stretch peak of CH_2_ was used to evaluate the lipid content of the tissue, in which a chemical map was created using the peak height at 2922 cm^–1^. The peak at 1080 cm^–1^ contains contributions from the phosphate symmetric stretch modes of phospholipids and nucleic acids as well as the C–O stretch and ring vibrations of carbohydrates. The peak at 1230 cm^–1^ includes phosphate asymmetric stretch and amide III modes. The maps showing the relative intensities of these bands were created from integrated peak areas from 1130 cm^–1^ to 1014 cm^–1^ and from 1275 cm^–1^ to 1205 cm^–1^, respectively [23]. The amide I band is particularly sensitive to changes in the secondary structure of proteins. The secondary structure of proteins was detected based on the amide I band located between 1700 cm^–1^ and 1600 cm^–1^ (baseline 1700 cm^–1^ to 1600 cm^–1^). Secondary derivative calculations were applied to estimate the number and position of the component bands. Based on these parameters, a multiple Gaussion curve-fitting process was performed to quantity the area of each component. The relative percentage of the secondary structural elements was obtained from the area under the Gaussion curve. This structure was identified based on the location of the maximum peak or the crest of the main peak: amide I maxima between 1660 cm^–1^ and 1650 cm^–1^ are generally assigned to α-helix; 1650 cm^–1^ to 1640 cm^–1^, random coil; 1640 cm^–1^ to 1620 cm^–1^, β-sheet; 1660 cm^–1^ to 1670 cm^–1^, 3_10_-helix; and approximately 1675 cm^–1^ to 1695 cm^−1^, anti-parallel pleated sheets/β-turns. The amide I region should be carefully assigned to a particular secondary structure or to determine the protein quantity [24].

### Statistical Analysis

SPSS 18.0 (USA) was used for statistical analysis. Data were compared with one-way ANOVA and repetitive-measurement ANOVA. The results were presented as mean ± standard error of the mean (SEM) and considered significantly different at p<0.05. The following values were considered statistically significant:*p<0.05, **p<0.01 vs. control; ^#^p<0.05, ^##^p<0.01 vs. model, *n* = 6 to 8.

## Results

### EGCG3”Me Inhibits the Formation of Reactive Carbonyl Compounds but not Blood Glucose

Figure 1 shows the structures of EGCG and EGCG3”Me. EGCG has flavan-3-ol structure with A and B rings and a D-galloyl group. EGCG3”Me contains a methyl ether group at the 3″ position of the D ring.

**Figure 2:**
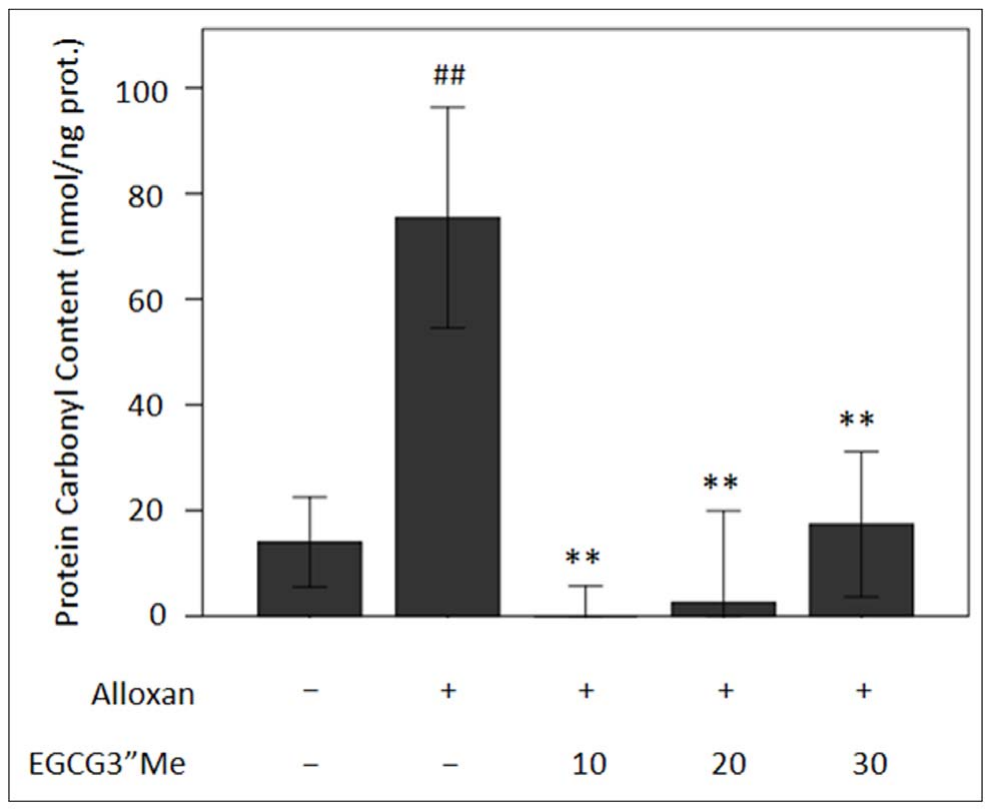
Inhibitory effect of EGCG3”Me on the formation of carbonylated proteins in the serum. ^##^represents statistical significance vs. control group; **represents statistical significance vs. diabetes model, n = 6–8.

Renal accumulation of AGEs, one of the RCCs, has been associated with the progression of diabetic nephropathy [25]. Carbonyl scavengers prevent carbonyl stress by inhibiting the formation of protein cross-links. Various AGE inhibitors have been developed, but only few carbonyl scavengers have been investigated based on ALEs-mediated effects [9]. To analyze EGCG3”Me and its neutralizing effect on RCCs contents in vivo, we detected the total carbonyl protein content in serum and 4-HNE protein extracted from a mouse kidney. Figure 2 shows that the serum carbonyl content was 75.45 nmol/ng protein in the diabetes model group and increased by 5.39 times (p<0.01) higher than that in the native control group. Different doses of EGCG3”Me administered to different groups significantly inhibited such changes, particularly for the low-dose group (10 mg/kg/d). In addition, carbonyl protein was absent in the serum.

**Figure 3:**
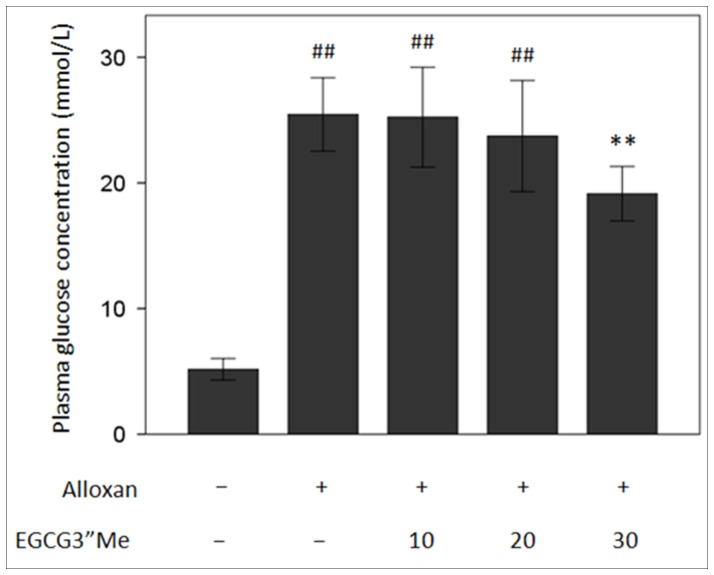
Effects of EGCG3”Me extracts on the blood glucose of diabetic mice. ^##^ represents the statistical significance vs. control group; **represents the statistical significance vs. diabetes model, n = 6 to 8.

After alloxan was intraperitoneally injected, the blood glucose concentration was significantly increased (25.45 mmol/L) compared with the control group. This value is 7 times greater than that of the control group (p<0.01). EGCG3”Me treatment groups, at the high-dose group, significantly lowered the blood sugar level (p<0.05). Both low- and medium-dose EGCG3”Me groups elicited no significant effect on blood glucose (Figure 3); therefore, the protective effect of low and medium doses of EGCG3”Me on diabetic mice is not attributed to the hypoglycemic effect.

**Figure 4:**
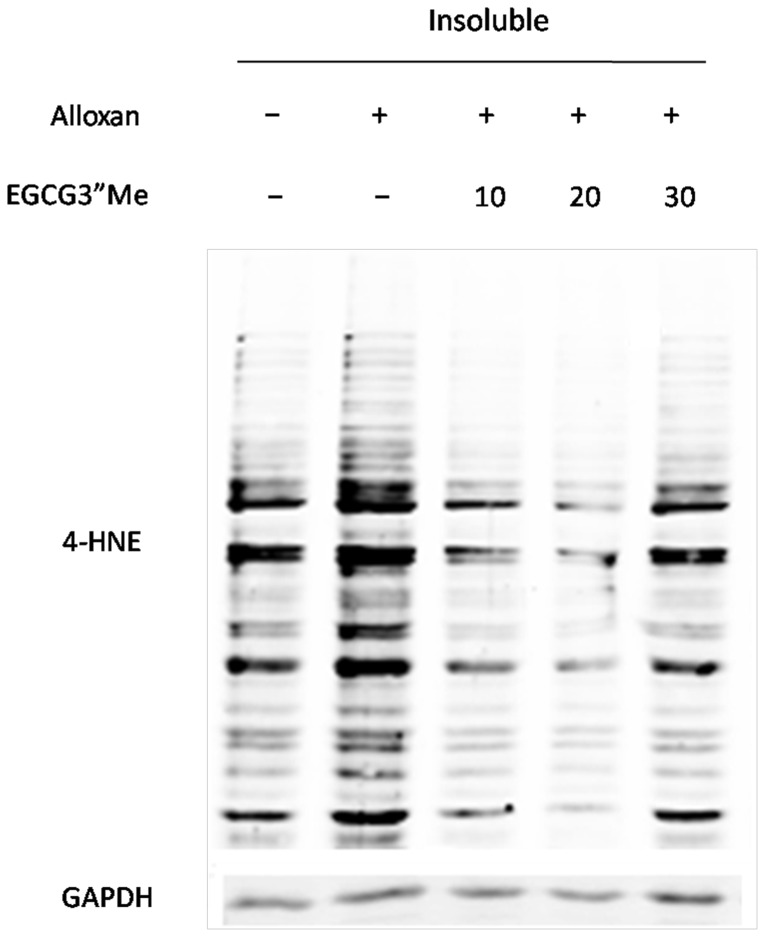
Immunoblotanalysis with anti-4-HNE antibodies to detect carbonylated proteins in the insoluble proteins SDS fraction (insoluble proteins) in the kidneys. EGCG3”Me treatment could greatly decrease the 4-HNE proteins content in the diabetic kidneys.

The signal intensities of 4-HNE were quantified by densitometry (normalized to total protein), indicating that changes in the levels of 4-HNE were not detected (data not shown) in the soluble protein fraction (Triton X-100 extraction); the changes in the levels of 4-HNE in the insoluble protein fraction (SDS extraction) as showed in Figure 4, 4-HNE modified proteins in diabetic kidney increased (approximately 1.98-fold) compared with the control counterparts (p<0.05). EGCG3”Me treatment could greatly decrease the 4-HNE proteins content, and at 10 mg/kg, the protein carbonyl content was approximately 0.27-fold lower than that in the diabetic kidney (p<0.05).

### Immunoblot Analysis of Ubiquitinated, Aggregated Protein in Different Treatments

**Figure 5:**
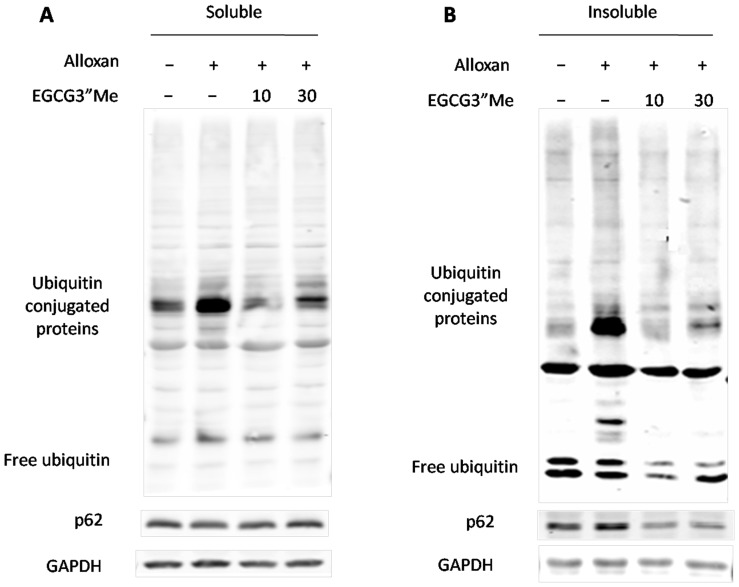
Immunoblot analysis of ubiquitin-conjugated proteins and p62 from the kidneys of diabetic mice. Ubiquitin-conjugated proteins profiles and p62 proteins can be used as a screening or diagnostic tool to characterize protein aggregates occurring in complex tissues and cells. (A) the levels of p62, free ubiquitin, ubiquitin-conjugated proteins with high molecular weights were not altered significantly for any treatment group. (B) p62 and insoluble ubiquitin modification proteins were highly expressed in the SDS fraction (insoluble proteins) in diabetic kidneys; EGCG3”Me treatment could evidently decrease this expression.

To determine whether or not alloxan-induced diabetes alters the ubiquitination pathway and develops substantial levels of insoluble proteins, we performed immunoblot analysis of the kidney proteins. In the soluble protein fraction (Triton X-100 extraction), changes in the levels of free ubiquitin were not detected, but a slight increase in ubiquitin levels was observed in alloxan-induced diabetes. Moreover, we did not detect changes in the levels of p62 (Figure 5A), which is involved in the delivery of ubiquitinated protein aggregates to autophagosomes. A sharply increase was observed in the levels of ubiquitinated proteins and p62 proteins with high molecular weights in the insoluble protein fraction of alloxan-induced diabetes. By contrast, EGCG3”Me treatment could decrease the level of ubiquitinated and aggregated proteins to a great extent, reaching values lower than those of the negative control group (Figure 5B).

### EGCG3”Me Treatment Decreases the Content of Anti-parallel β-sheet/aggregated Strands and Lipid Content in the Diabetic Kidney

**Figure 6:**
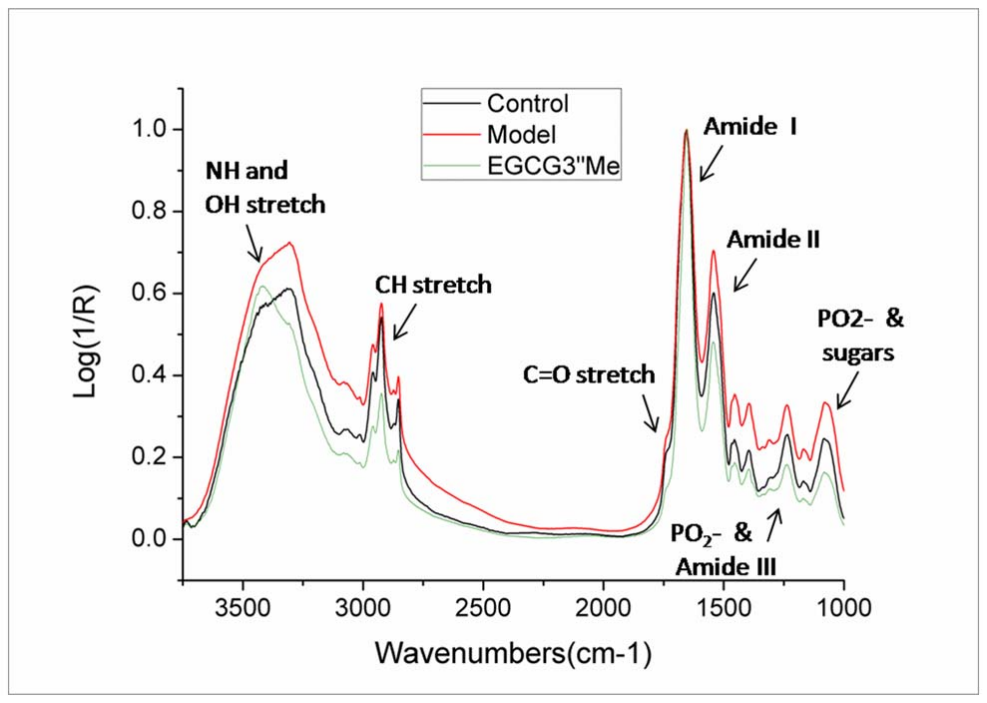
Kidney tissues morphologies were detected in processed IR spectroscopic maps. IR spectra of control group (black), model group (red), and EGCG3”Me-treated group (green) normalized to the amide I band. Arrows indicate the relevant phospholipid peaks of the C–H stretch band at 2922 cm^–1^, the C = O stretch at 1740 cm^–1^, and the phosphate bands at 1080 and 1240 cm^–1^ in the model and decreased by EGCG3”Me, which indicated that EGCG3”Me decreasing lipid and other cell content.

Infrared spectrometry techniques are extremely valuable to study structural protein modifications and the changes in the relative proportion of tissue components based on their spectral signatures without the use of stains or other probes. Therefore, chemical compositions and secondary protein structural changes can be subjected to correlational analysis, thereby providing insights into the disease process without additional mechanisms [26]. We examined the distribution of absorbance peaks in the major tissue components (Figure 6). The correlation between the chemical maps based on the intensity of peaks at the CH stretch as well as at 1080 and 1230 cm^–1^ indicated the phospholipid levels increased in the diabetic kidney [23]. By contrast, the phospholipid remained low in control group, especially in EGCG3”Me treatment group. This result was confirmed by a decrease in other distinct lipid peaks, including the C = O stretch at 1740 cm^–1^. EGCG3”Me could decrease lipid level significantly.

**Figure 7:**
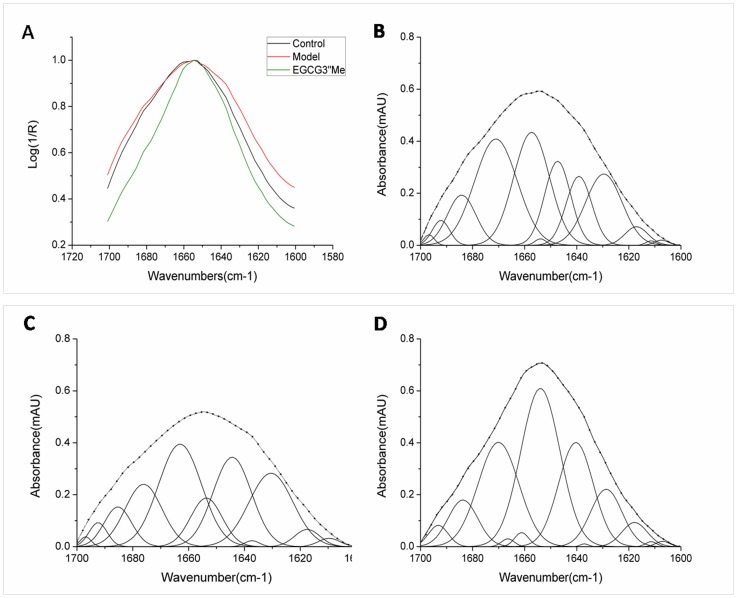
EGCG3”Me treatment could sharply decrease the formation of antiparallel β-sheets in diabtetic kidneys. FTIR spectra of amide I (normalization and appropriate baselines from 1700 cm^–1^ to 1600 cm^–1^) show that alloxan-induced diabetic model correspond to a high β-sheet content (1675 cm^–1^ to 1695 cm^−1^); EGCG3”Me increased α-helix content (1660 cm^–1^ and 1650 cm^–1^)(a), and the different secondary structure bands using Gaussian curve fitting program Origin 8.0 ((B), control; (C), model; (D), EGCG3”Me treatment).

The intensity of the amide I peak of the diabetic mice was evidently decreased and considered as another strikingly consistent factor. In the EGCG3”Me treatment, the intensity of the amide I peak of the EGCG3”Me treatment group was increased compared with that of the negative control group (Figs. 6 and 7A).

To understand the changes in protein structure, we quantitatively analyzed the secondary structure of the amide I band. This structure can be investigated based on C = O vibrations of the protein backbone (amide I region, baselines from 1600 cm^–1^ to 1700 cm^–1^), in which the characteristic β-sheet structure can be distinguished from other structures such as a random coil and α-helix. In Figure 7, the secondary structures of the α-helixes of the control group, the diabetic model, and the EGCG3”Me treatment group were 23.27%, 8.91%, and 34.18%, respectively, whereas the antiparallel β-sheet/aggregated strands were 11.13%, 22.74%, and 9.63%, respectively. This result indicated that EGCG3”Me treatment could protect the proteins in a diabetic kidney from structural changes.

## Discussion

IUP profiles and p62 proteins can be used as a screening or diagnostic tool to characterize genetic and age-dependent factors that alter the long-term function of autophagy and clearance of protein aggregates occurring in complex tissues and cells [4], [27]. The results of this study indicated that a significant fraction of p62 and ubiquitinated protein aggregates is present in an insoluble conformation in diabetic kidneys, the mechanism of which maybe come from carbonyl stress-mediated formation in the early development of diabetic nephropathy. Glycation induced protein aggregation has been implicated in the development of diabetic complications [28]. Previous studies showed that RCCs, such as AGEs and ALEs, are strong electrophiles exhibiting high reactivity with Cys, His, and Lys nucleophile residues [29]. Studies have further indicated that 4-HNE is related to the formation of IUP aggregates. The extensive modification of cellular proteins by 4-HNE and related aldehydes leads to the formation of protein aggregates that accumulate in the cells and are not degraded by proteasomes. 4-HNE- and peroxynitrite-induced protein modification associated with the defect in the ubiquitin-proteasome system results in the accumulation of modified proteins and contributes to cell death [10]. Thus far, most of the carbonyl stress inhibitors used have been developed to prevent the accumulation of AGEs in diabetes; however, only a few carbonyl scavenger agents known to reduce accumulation have been tested in vivo on the progression of diseases.

Our study showed that the formation of carbonyl protein in the serum and 4-HNE was evidently increased in insoluble protein in alloxan-induced diabetic kidneys. Furthermore, EGCG”3Me could greatly impede the progress of diseases. EGCG3”Me is one of the tea-derived *O*-methylated catechins could evidently decrease the accumulation of p62 and IUP in diabetic kidneys; therefore, EGCG3”Me could decrease the amounts of ubiquitinated, modified protein aggregates in alloxan-induced diabetes, which indicated that EGCG3”Me could block carbonyl stress-mediated formation of ubiquitinated, modified protein aggregates in the early development of diabetic nephropathy. Moreover, EGCG3”Me was more effective than EGCG (data not shown).

Our previous FT-IR study demonstrated that the carbonyl-amino cross-links contribute to a sharp decrease in intensity of the amide I band, whereas β-sheet increases evidently [8]. β-sheet structure is the “seed” of misfolded aggregates [6]. Our studies indicated that carbonyl stress is essentially the same as the formation of anti-parallel β-sheet and phospholipids of cell content increased in diabetic kidneys. In addition, EGCG3”Me treatment could sharply decrease the anti-parallel β-sheet/aggregated strands in a diabetic kidney and increase the α-helix content when compared with the diabetic mouse group. The membrane embedded in the α-helix maybe important for the same molar absorptivity as a water-soluble helix [24], which maybe is related to the cell nutrient uptake function.

On the other hand, FT-IR study revealed that the phospholipid and other lipid content increased in early renal injury of diabetic kidneys. Recent evidences have suggested that β-sheet peptides can self-organize into oligomeric subunits with high toxicity potential in the simulated lipid environments [30]–[32]. Our studies indicated that, the carbonyl stress may contribute to the cell content levels, causing carbonyl-animo crosslinks resulting in lipid peroxidization, which formation lipofuscin or other aggregations.

**Figure 8:**
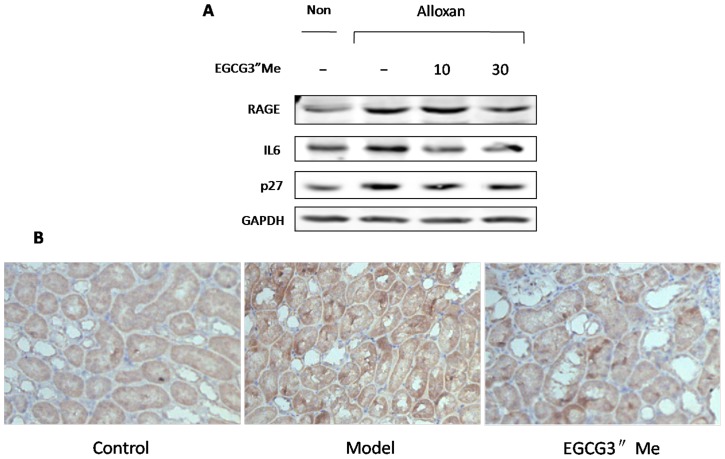
EGCG3”Me decrease RAGE, IL6, p27 protein levels in diabetic kidney. (A), Western blot analysis of RAGE, IL6, and p27 expression was carried out in diabetic kidney compared with control; (B), Immunochemistry analysis of RAGE (brown) in diabetic kidney, which indicating that EGCG3”Me inhibited the development of diabetic nephropathy and other complications of diabetes.

RCCs leads to a positive feedback cycle to sustain elevated the receptor for advanced glycation end products (RAGE) expression. Moreover, because increased receptor levels at the cell surface will promote preassembly, RAGE increase the aggregates formation and accumulation [33]. This would explain the hyperactivation of the RAGE pathway in the development of chronic inflammatory and other degenerative disorders. For example, the vicious cycle in a hyperglycemic state results in the development of diabetic vascular complications. In addition, frequent hypoglycemia attacks in patients treated with oral hypoglycemic drugs or insulin injection can produce RAGE, which may exhibit a higher affinity to AGE ligands, resulting in a higher susceptibility to diabetic complications [34]. RAGE is involved not only in complications of diabetes but also in type I and II diabetes [35]. The current study showed that EGCG3”Me treatment could decrease the expression of RAGE (Figure 8), which not come from the hypoglycemic effect, but mainly come from the anti-carbonyl stress effect. Given that the cell cycle is regulated by kinases and phosphatases, which are controlled at the level of ubiqutin-dependent proteolysis, the conditions leading to aggregate formation likely cause cell cycle disregulation; as a result, an accelerated rate of aging process of the organs is observed [36]. p27 exhibits several hallmarks: as a negative regulator of G1 progression and a mediator of TGF-beta-induced G1 arrest, and EGCG3”Me treatment could decrease the expressions of p27 and inflammation-related IL6 in diabetic kidneys compared with diabetic mice (Figure 8). All the results indicated that EGCG3”Me inhibited the development of the diabetic complications.

In conclusion, the present study identified a novel mechanism by which carbonyl-stress-induced IUP aggregates contributed to the development of diabetic nephropathy. In addition, galloyl D ring tea catechins, particularly EGCG3”Me, can effectively reverse the process to stave off the progression of diabetes nephropathy at early intervention. However, effective treatments remain unavailable at the present time because of refractory diabetic complications. Our results further indicated that the generation of drugs inhibiting the formation of carbonyl-stress-induced IUP aggregates may represent a new therapeutic strategy to retard the progression of diabetic nephropathy and other diabetic complications.
